# A Novel Rapid Sample Preparation Method for MALDI-TOF MS Permits *Borrelia burgdorferi* Sensu Lato Species and Isolate Differentiation

**DOI:** 10.3389/fmicb.2020.00690

**Published:** 2020-04-21

**Authors:** Anna-Cathrine Neumann-Cip, Volker Fingerle, Gabriele Margos, Reinhard K. Straubinger, Evelyn Overzier, Sebastian Ulrich, Andreas Wieser

**Affiliations:** ^1^Division of Infectious Diseases and Tropical Medicine, University Hospital LMU, Munich, Germany; ^2^German Center for Infection Research (DZIF), Partner Site Munich, Munich, Germany; ^3^National Reference Center for Borrelia, Bavarian Health and Food Safety Authority, Oberschleissheim, Germany; ^4^Chair of Microbiology and Mycology, Department of Veterinary Sciences, Faculty of Veterinary Medicine, Ludwig Maximilian University of Munich, Munich, Germany; ^5^Chair of Medical Microbiology and Hospital Epidemiology, Faculty of Medicine, Max von Pettenkofer Institute, Ludwig Maximilian University of Munich, Munich, Germany

**Keywords:** *Borrelia burgdorferi* sensu lato, MALDI-TOF MS, typing, sample preparation, MALDI-TOF MS library, strain typing, automatic identification

## Abstract

The genus *Borrelia* comprises vector-borne bacterial pathogens that can severely affect human and animal health. Members of the *Borrelia burgdorferi* sensu lato species complex can cause Lyme borreliosis, one of the most common vector-borne diseases in the Northern hemisphere. Besides, members of the relapsing fever group of spirochetes can cause tick-borne relapsing fever in humans and various febrile illnesses in animals in tropical, subtropical and temperate regions. *Borrelia* spp. organisms are fastidious to cultivate and to maintain *in vitro*, and therefore, difficult to work with in the laboratory. Currently, borrelia identification is mainly performed using PCR and DNA sequencing methods, which can be complicated/frustrating on complex DNA templates and may still be relatively expensive. Alternative techniques such as matrix-assisted laser desorption/ionization time-of-flight mass spectrometry (MALDI-TOF MS) are not well established for *Borrelia* spp., although this technique is currently one of the most used techniques for rapid identification of bacteria in microbiological diagnostic laboratories. This is mainly due to unsatisfactory results obtained by use of simple sample preparation techniques and medium-contamination obscuring the mass spectra. In addition, comprehensive libraries for *Borrelia* spp. MALDI-TOF MS have yet to be established. In this study, we developed a new filter-based chemical extraction technique that allows measurement of high quality *Borrelia* spp. spectra from less than 100,000 bacteria per spot in MALDI-TOF MS. We used 49 isolates of 13 different species to produce the largest mass-library for *Borrelia* spp. so far and to validate the protocol. The library was successfully established and identifies >96% of used isolates correctly to species level. Cluster analysis on the sum spectra was applied to all the different isolates, which resulted in tight cluster generation for most species. Comparative analysis of the generated cluster to a phylogeny based on concatenated multi-locus sequence typing genes provided a surprising homology. Our data demonstrate that the technique described here can be used for fast and reliable species and strain typing within the borrelia complex.

## Introduction

The genus *Borrelia* comprises tick-borne spirochetal bacteria that are maintained in natural transmission cycles by vector ticks and reservoir hosts. The genus is divided into several clades including relapsing-fever group of spirochetes, the Lyme borreliosis group, reptile-associated *Borrelia* species and a recently described echidna-associated species detected in Australia (Loh et al., 2016; Gofton et al., 2018; Margos et al., 2019). The bacteria are of medical and veterinary importance as several species can cause disease in humans and animals such as relapsing fever, Lyme borreliosis or avian spirochetosis (McNeil et al., 1948; Parola and Raoult, 2001). The distribution range of these parasitic bacterial species depends on the presence of their vector ticks and vertebrate reservoir hosts. The species in the Lyme borreliosis group are vectored by several tick species of the genus *Ixodes* and utilize rodent, avian and reptile reservoir hosts (Piesman and Gern, 2004; Margos et al., 2011; Radolf et al., 2012). Accordingly, their geographic distribution is mainly between 40- and 60-degree latitude where Lyme borreliosis represents the most frequently reported vector-borne disease in Europe and the United States of America. For example, the number of new infections in the United States is estimated to be >300,000 each year whilst in Europe the number of new cases is estimated to exceed 200,000 per year in Germany alone based on health insurance data (Steere, 2001; Stanek and Strle, 2009; Muller et al., 2012; Schwartz et al., 2017).

The *Borrelia burgdorferi* sensu lato (s.l.) species complex (Lyme borreliosis group of spirochetes) currently comprises >20 named genospecies, six of which are regularly associated with human disease. Of these, five occur in Europe including *B. afzelii*, *B. bavariensis, B. burgdorferi* sensu stricto (s.s.), *B. garinii*, and *B. spielmanii*, while two, *B. burgdorferi* s.s. and the recently discovered *B. mayonii*, occur in the United States (Pritt et al., 2016; Lohr et al., 2018). Species and strain structure are difficult to ascertain based on single locus molecular analysis and require multi-locus sequence analyses (MLST/MLSA) (Margos et al., 2012), which may be a costly exercise.

Generally, the diagnosis of bacterial pathogens of medical and veterinary importance requires fast, reliable, and economical methods. Matrix-assisted laser desorption/ionization time-of-flight mass spectrometry (MALDI-TOF MS) has emerged as the tool of choice for the rapid identification of various cultured bacterial and fungal pathogens. Routine MALDI-TOF MS has been established in clinical microbiology laboratories almost worldwide due to its excellent performance and ease of use (Seng et al., 2009; Bizzini and Greub, 2010; Croxatto et al., 2012; Wieser et al., 2012; Singhal et al., 2015; Ulrich et al., 2016; Tracz et al., 2018). As this method is based on comparing the protein profile of unknown microorganisms with a database in routine use, it is required to establish a good spectral database for species and strains and to develop a reliable sample preparation protocol (Fotso Fotso et al., 2014).

For many gram-negative bacteria, such as Enterobacterales, MALDI-TOF MS is well established and many million bacterial cells from culture are smeared directly onto the target and co-crystallized with the matrix (Wieser et al., 2012). For borrelia cultures, such procedures are not applicable due to its growth in liquid medium and requirement of very protein rich, complex medium (Preac-Mursic et al., 1986). MALDI-TOF MS has been explored for the detection of vector-borne pathogens and simultaneous identification of vector species (Yssouf et al., 2014; Yssouf et al., 2016; Boyer et al., 2017). Whilst in some cases the technology offered exciting advantages over other methods, it remained unsuccessful in others. Previous investigations have shown that MALDI-TOF MS is able to distinguish several of the known *Borrelia* species including the most prevalent Lyme borreliosis- causing species in Europe such as *B. afzelii*, *B. garinii*, *B. burgdorferi* s.s., and *B. spielmanii* (Calderaro et al., 2014; Fotso Fotso et al., 2014). While these studies demonstrated the general potential of MALDI-TOF MS for identification of *Borrelia* spp., a comprehensive library taking into account intra-species variation and a protocol offering high sensitivity and user friendliness is still missing.

Current protocols to isolate proteins for spectra of *Borrelia* spp. require large amounts of bacterial culture and are not very sensitive. This is mainly due to unsatisfactory results obtained when simple sample preparation techniques are used, because medium components stick to the bacteria and obscure the bacterial signature, which creates difficulties in rendering *Borrelia* spp. proteins accessible to analysis after simple on-chip extraction. In addition, insufficient amounts of proteins from small numbers of bacterial cells make it problematic to yield reliable spectra. Furthermore, comprehensive libraries for *Borrelia* spp. MALDI-TOF MS are not available.

Here, we report the development of an improved sample preparation method for borrelia organisms and the establishment of a reference database that can be used directly and can serve as a basis for further development. Our data show that MALDI-TOF MS used with this novel preparation protocol is a cost effective, rapid and reproducible tool to measure protein signatures sensitively. Apart from species identification and strain typing, this novel protocol for protein extraction from *Borrelia* spp. may allow for other uses such as proteomics or phylogenic studies.

## Materials and Methods

### General Considerations

The isolates used within this study are potential human pathogens, thus all experiments and extraction protocols have been performed under biosafety precautions applicable for class 2 organisms (BSL 2). This includes that all steps handling open cultures of the organisms are performed in class 2 laminar flow hoods. All reagents used including the buffer solutions and water are sterilized and free of DNA/RNA or other contaminants. All reagents and media have been tested for sterility prior to use. The workup of clinical samples would also require the handling under BSL 2 conditions.

### Bacterial Isolates and Cultivation

*Borrelia* spp. and isolates included into the study, source, country of origin, year of isolation and multilocus sequence type (MLST ST) are given in Table 1. *Borrelia* spp. isolates were cultured either in commercial Barbour-Stoenner-Kelly-H [BSK-H (Sigma Aldrich, Germany] or using MKP medium and conditions described previously (Barbour, 1984; Preac-Mursic et al., 1986; Pollack et al., 1993). Briefly, cultures were kept in 7 mL liquid MKP medium supplemented with 6% heat-inactivated rabbit serum (Sigma-Aldrich) at 33°C. Cultures were harvested when bacteria growth was in the exponential phase. For testing different media some spirochetes of the *B. burgdorferi* s.l. complex (*B. burgdorferi* s.s., *B. garinii*, and *B. bavariensis*) were grown in 6 mL of BSK-H at 33°C and 5% CO_2_. For molecular analyses, genomic DNA was extracted via a Maxwell^®^ 16 using a Maxwell LED DNA kit (Promega, Germany). MLST data were extracted from the MLST database at https://pubmlst.org/borrelia/.

**TABLE 1:** *Borrelia* spp. isolates included in the study.

**Isolate**	**Species**	**Biological origin**	**Country of origin**	**Year of isolation**	**MLST ST**
PBabu	*B. afzelii*	Human	Germany	2001	71
PBas	*B. afzelii*	Human	Germany	1989	80
PDrP	*B. afzelii*	Human	Germany	1988	463
PHa	*B. afzelii*	Human	Germany	1992	71
PHot	*B. afzelii*	Human	Germany	1987	nd
PKuk	*B. afzelii*	Human	Germany	1998	71
PLud	*B. afzelii*	Human	Germany	1988	463
PNad	*B. afzelii*	Human	Germany	1988	470
PBaeII	*B. bavariensis*	Human	Germany	1990	84
PBi	*B. bavariensis*	Human	Germany	1984	84
PBN	*B. bavariensis*	Human	Germany	1999	84
PFin	*B. bavariensis*	Human	Germany	1991	84
POb	*B. bavariensis*	Human	Germany	1993	85
PShb	*B. bavariensis*	Human	Germany	1988	nd
PTrob	*B. bavariensis*	Human	Slovenia	1988	85
DN127	*B. bissettiae*	Tick	United States	1985	272
PGeb	*B. bissettiae*	Human	Germany	1996	667
B31	*B. burgdorferi* s.s.	Tick	United States	1981	1
PAli	*B. burgdorferi* s.s.	Human	Germany	1994	1
PBoe	*B. burgdorferi* s.s.	Human	Germany	2002	1
PDri	*B. burgdorferi* s.s.	Human	Germany	1988	24
PFi_I	*B. burgdorferi* s.s.	Human	Germany	1985	284
PGl	*B. burgdorferi* s.s.	Human	Germany	1993	21
PHas	*B. burgdorferi* s.s.	Human	Germany	1992	1
PKaII	*B. burgdorferi* s.s.	Human	Germany	1984	1
PLue	*B. burgdorferi* s.s.	Human	Germany	1999	3
PMi	*B. burgdorferi* s.s.	Human	Germany	1994	20
CA446	*B. californiensis*	Rodent	United States	1995	447
SCW22	*B. carolinensis*	Bird	United States	1994	450
PBr	*B. garinii*	Human	Germany	1985	244
PCoo	*B. garinii*	Human	Germany	1987	573
PFe	*B. garinii*	Human	Germany	1992	244
PHei	*B. garinii*	Human	Germany	1987	246
PKi	*B. garinii*	Human	Germany	1992	245
PKuf	*B. garinii*	Human	Germany	1994	251
PLa	*B. garinii*	Human	Germany	1988	245
PNov	*B. garinii*	Human	Germany	1990	180
PRef	*B. garinii*	Human	Germany	1989	482
PWudII	*B. garinii*	Human	Germany	1988	nd
HO14	*B. japonica*	Unknown	Japan	nd	453
25015	*B. kurtenbachii*	Tick	United States	nd	280
PotiB2	*B. lusitaniae*	Tick	Portugal	nd	456
PoTiB3	*B. lusitaniae*	Tick	Portugal	1993	766
PHap	*B. spielmanii*	Human	Germany	1989	159
PMEW	*B. spielmanii*	Human	Germany	1987	159
PSig2	*B. spielmanii*	Human	Germany	2000	nd
IST7	*B. turcica*	Tick	Turkey	2003	793
S21-Z1	*B. turcica*	Tick	Greece	2017	nd
VS116	*B. valaisiana*	Tick	Switzerland	nd	95

*nd, no data.*

### MALDI-TOF MS Sample Preparation

Two sample preparation protocols were tested. One protocol was based on the “ethanol formic acid extraction” protocol (Bruker Daltonics) and previous studies (Rettinger et al., 2012) (see detailed extraction protocol in Supplementary Material). This extraction method was found to be insensitive and insufficient regarding the removal of media components (see Supplementary Figure S1). Therefore, a second protocol was developed, which yielded data that are more sensitive and was subsequently used for the generation of the library and cluster analysis.

### Newly Developed Extraction Protocol

Cultures were grown to a density of approximately 10^6^ organisms per ml. Bacterial growth density was determined using the method described earlier (Hepner et al., 2019). Briefly, 13 μL of borrelia culture were applied on a glass slide covered with a cover slip of 76 × 26 mm. 50 fields of view were counted and the density of borrelia calculated using the formula: number of bacteria x 3500 × 1000/number of fields of view × 3. To prevent media artifacts in measurements, cultures were rigorously washed before protein preparation. For this, 1.5 mL of the culture were centrifuged in a microcentrifuge at 9168 × *g* (10,000 rpm) for 20 min at RT to harvest the bacteria. The supernatant was discarded and the cell pellet resuspended in 500 μL of phosphate buffered saline (PBS, 8 g NaCl, 0.2 g KCl, 1.78 g NaH_2_PO_4_⋅2H_2_O, 0.24 g KH_2_PO_4_, ad 1 L dest. H_2_O, pH 7.4). The washing procedure was repeated four times and before each washing procedure, the bacterial solution was drawn through a cannula (Ø 0.45 × 25 mm) 20 times to separate the bacteria, thereby enabling an optimal removal of media components. Finally, the pellet was resuspended in 500 μL of fresh PBS solution. Immobilization, washing and extraction of spirochete organisms on GHP filter membranes was proceeded as recently published (Neumann et al., 2019). The technique was slightly modified. In brief, 300 μL of washed borrelia organism suspension, or pure media as a control, were applied into one well of a GHP 0.2 μm pore size 96-well filter plate (Pall, Port Washington, NY, United States) using vacuum suction. The membranes with immobilized bacteria were washed three times for 5 min with 300 μL dest. H_2_O. Here again, vacuum suction was used to avoid any losses of cell mass during washing. Afterward, the filter plate was placed on ice, and 100 μL of ice-cold acetone was added and incubated for 5 min. After removing the acetone by centrifugation or vacuum suction, residual acetone was evaporated by incubation at 37°C for 7 min. Elution of the extracted proteins was performed by adding 20 μL elution solution [50% acetonitrile, 35% formic acid, 15% dest. H_2_O (v/v)] to each well. After 5 min of incubation at room temperature, a 96-well collection plate was placed below the filter plate and the elution solution was centrifuged or vacuum-sucked through the filter membrane into the collection plate [1467 × *g* (4000 rpm), 3 min at 20°C].

### MALDI-TOF MS Analysis

The extracts were directly spotted in triplicates (1 μL each of the 20 μL eluate) onto three polished steel MSP-96 MALDI targets (Bruker Daltonik GmbH, Bremen, Germany). Spots were overlaid with 1 μL α-cyano-4-hydroxy-cinnamic acid [HCCA, 10 mg/mL in 50% acetonitrile, 47.5% dest. H_2_O and 2.5% trifluoroacetic acid, (v/v)]. The classic layering protocol, which is standard for bacterial identification as described here, was found to be reliable and robust. Compared to pre-mixing of matrix and extracts or re-crystallization with acetone no important differences were observed. After matrix drying, the measurements were performed on a Microflex LT benchtop mass spectrometer equipped with a 60 Hz nitrogen laser and controlled by FlexControl software (version: 3.4.135.0, Bruker Daltonik GmbH, Bremen, Germany). Spectra were recorded in the positive linear mode. The parameter settings were optimized for the mass range between 2000 and 20,000 Da and were as followed: ion source 1: 0 kV, ion source 2: 18.25 kV, pulsed ion extraction time: 130 ns. Gain and laser power were used as they are standard for bacterial identification and recommended by the manufacturer. For instrument calibration an external standard was chosen consisting of masses in the range between 3637.8 and 16952.3 Da (bacterial standard, BTS, Bruker Daltonik GmbH, Bremen, Germany). All acquisitions were recorded automatically in the instrument software and based on averaging 240 satisfactory shots in 40 shot steps (MBT_AutoX Algorithm).

### *Borrelia* MALDI-TOF-MS Database

For each of the 49 isolates (13 species), a minimum of 27 replicates (triplicate in three replicates each on three targets) were analyzed. The obtained spectra were visualized and analyzed by the FlexAnalysis software (version: 3.4, Bruker, Bremen, Germany). For visualization, smoothing by Savitzky-Golay-Filtering was applied, baseline subtraction was applied as well (built into the software). Single spectra with low intensity (<10^3^ arbitrary units/a.u.) were not included in the borrelia strain database, only spectra passing the automated QC algorithm as used in the AutoX-Algorithm (Bruker, Bremen, Germany) were included in the analysis. Based on these spectra, the reference Main Spectrum Profile (MSP) was generated using the automated function of the Biotyper Software (version 3.1, Bruker, Bremen, Germany). MSP creation was performed by extracting information about peak position, intensity and frequency by an unbiased algorithm, as recommended by the Bruker Daltonics Guidelines. The obtained MSP spectra were used for the borrelia database generation.

For cluster analysis (MSP dendrogram) the Biotyper MSP Creation Standard Method was used and the following settings were chosen: “Distance Measure Correlation”, “Linkage Centroid,” “Max. Number of Top Level Nodes 0,” “Score Threshold Value for Single Organism 500,” “Score Threshold Value for Related Organism 0.” The linkage function was normalized in the range between 0 (perfect match) and 1000 (no match).

### *Borrelia* spp. Database Validation

All spectra were individually matched to the database to obtain score values for identification. Measurements were performed according to the AutoX protocol used for routine bacterial identification with the Biotyper package on a Microflex LT MALDI-TOF (Bruker). The results were recorded for all 1,438 sum spectra each consisting of sums of about 240 laser shots which is automatically performed using the common AutoX protocol.

### Phylogeny of MLST Data

Sequence data for isolates included in this study available at the MLST database^1^ were downloaded and used for phylogenetic analysis (Table 1). Data can be accessed via the website either using the isolate name or the ST number. A maximum likelihood phylogeny using concatenated sequences of eight housekeeping genes (Margos et al., 2008) was generated in MEGA5 (Tamura et al., 2011). Settings were as follows: the General Time Reversible (GTR) model was chosen as substitution model with 1,000 bootstrap replications for branch support. Rates among sites were uniform, gaps and missing data were deleted. The Nearest-Neighbor-Interchange (NNI) was used as heuristic method. Codons included were 1st+2nd+3rd+Non-Coding.

## Results

We evaluated different extraction techniques for analysis of *Borrelia* spp. with MALDI-TOF MS. Initially, an established extraction protocol (detailed method in Supplementary Material) was applied to 20 samples of Borrelia from three species. Sum spectra of three different *Borrelia* spp. showed heavy contamination with media components and required large cell mass (Supplementary Figure S1). The negative control consisted of BSK-H medium only and was extracted in the same way as borrelia cultures. The spectra of this negative control showed mass peaks, very similar to those created of media including *Borrelia* spp. in the mass range between 2,000 and 8,500 *m/z* (Supplementary Figure S1) suggesting that not all medium proteins had been removed by the classical extraction protocols.

**FIGURE 1:**
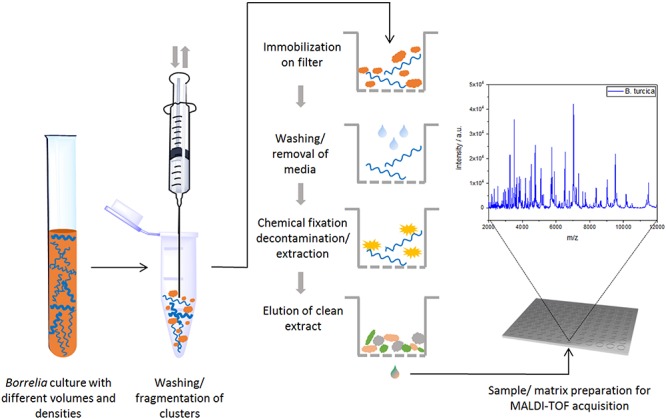
Schematic workflow for sample preparation (and MALDI-TOF MS measurement) for *Borrelia* spp. isolates.

**FIGURE 2:**
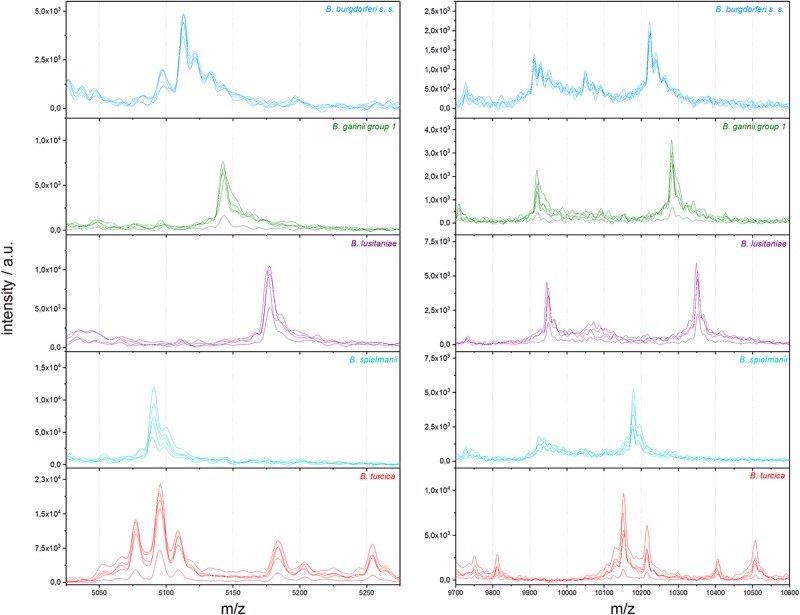
Representative differences in MALDI-TOF MS profiles on species scale for *B. burgdorferi* s.s., *B. garinii* group 1, *B. lusitaniae*, *B. spielmanii*, and *B. turcica*; each in the mass range between 5,025 and 5,275 *m/z* and 9,700 and 10,600 *m/z* (a.u., arbitrary units).

To eliminate medium artifacts we developed a new extraction protocol and evaluated its performance for analyzing *Borrelia* spp. isolates using MALDI-TOF MS. The extra washing steps included in the new extraction protocol (see Figure 1) purged much of the contaminating medium proteins leading to much cleaner and more reproducible protein spectra (Supplementary Figure S2). Indeed, medium only extractions using the filter based extraction techniques never passed the automated quality control algorithms due to lack of signals using the pre-set signal to noise cutoffs used for all measurements. They are therefore not presented. Multiple replicates were performed demonstrating unique spectral features of the different species as well as isolates (see Figure 2 and Supplementary Figure S2). Medium-only controls included in the study confirmed that medium peaks could be eliminated using the filter immobilization and washing technique. Limit of detection studies were performed to demonstrate the sensitivity of the newly developed protocol. Extracts of ≤100,000 bacteria cells per target spot were sufficient to produce multiple, consistent measurements. Amounts of >10^6^–10^7^ were also measured without problems or inhibition. The overall number of borrelia organism used to obtain 20 μL of protein elution solution was 1.5–2 × 10^6^ cells. Of the 20-μL eluate 1 μL was used for one spot. From each spot only a small fraction is analyzed/ablated by the laser, thus the sum spectra shown in Figure 2 and Supplementary Figure S2 are generated from spots containing extracts of way less than 100,000 cells. Overall, 49 different isolates of 13 different *Borrelia* species were extracted multiple times from different cultures to generate a representative mass library for identification of samples at the strain as well as species level. Overall, 1,438 sum spectra were generated. Spectra were found to be richest in peaks in the range between 2,000 and 20,000 *m/z*. Thus, this spectral mass range was chosen for the automated measurements and library generation.

**FIGURE 3:**
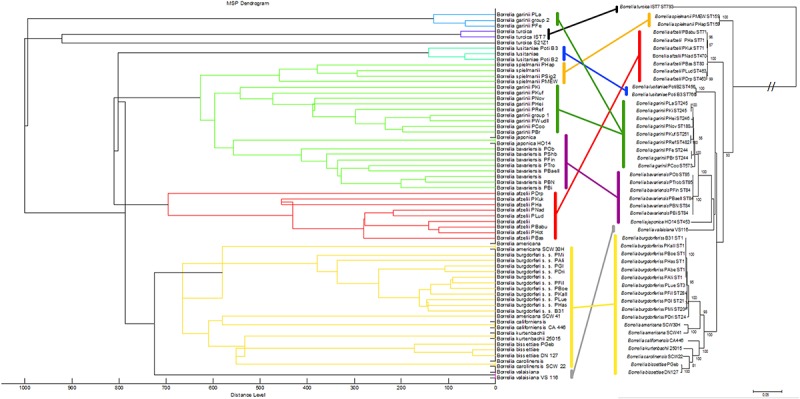
Comparison of the MALDI-TOF MS spectral clusters (left side) to the MLSA clustering (right side). Overall strong accordance of species clustering in genetic and MALDI-TOF MS data analyses were observed. Cluster analysis (MSP dendrogram) of the Main Spectrum Profiles (MSP) loaded into the *Borrelia* spp. strain library. The following settings were chosen for dendrogram generation: distance measure, correlation; linkage, centroid; max. number of top level nodes, 0; score threshold value for single organism, 500; score threshold value for related organism, 0.

We also compared the mass spectra of the individual isolates using cluster analysis: the relationships between the mass spectra of all isolates were assessed and visualized in a dendrogram (Figure 3, left side). Most *Borrelia* spp. isolates belonging to the same species clustered together in the dendrogram. An exception was *B. garinii*; the isolates of this species formed two different clusters; *B. garinii* isolates PLa and PFe formed a different clade (in this study termed *B. garinii* mass group 2) from the remaining *B. garinii* isolates (termed *B. garinii* mass group 1). The same isolates used for mass spectra cluster analysis were investigated using MLSA. A phylogenetic tree constructed with available concatenated sequences of the isolates showed clustering according to the species (Figure 3, right side). Although differences in topology were apparent between spectra cluster and sequence cluster, very similar clades were generated by both methods (linked with colored lines). The two *B. garinii* isolates, which formed the divergent cluster in the mass spectrum cluster analysis, clustered together with other *B. garinii* isolates in the genetic phylogeny (Figure 3).

**FIGURE 4:**
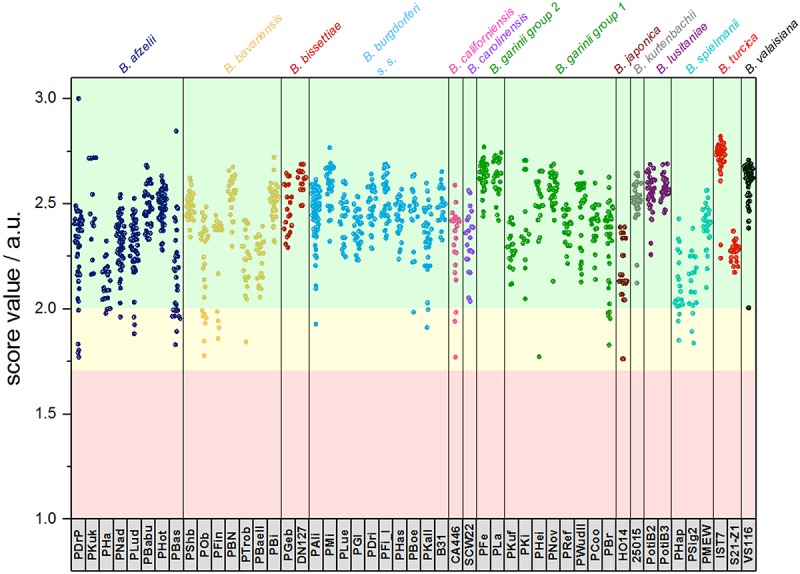
Typing results obtained from 1 sum spectrum each. Green: >2.0 secure species/isolate identification, yellow: range 1.7–1.999 genus level identification, red: range <1.7 no reliable identification. In this study, score values ≤1.7 were not obtained (a.u., arbitrary units).

For the assembly of the database, spectra of all isolates were used. First, average spectra for all isolates of each species were generated. Thereby, due to extensive differences in the mass spectra, *B. garinii* was separated in the analysis into *B. garinii* mass group 1 and 2, respectively, which were considered separate entities for the analysis, resulting in 14 groups/species. This yielded an overall correct species identification rate (score ≥ 2.0) of 85.5% over all species, while in average 8.6% of identifications were probable (score 1.7-1.999). The performance varied considerably between the species as well (Table 2). Second, all individual strain spectra were inserted into the database as separate entities. Matching with this library without any averaged species-spectra resulted in 96.0% correct species/strain identification (score ≥ 2.0), while 2.8% were probable (score 1.7-1.999), few measurements were unreliable. Finally, a database combining both approaches was used feeding the averaged species spectra as well as the individual strain spectra into the database. This resulted in an overall confident species identification of 96.5% over all species (score ≥ 2.0) and the remaining measurements to be probable identifications (score 1.7-1.999). Thereby, especially the average score value of those species with many different isolates in the analysis increased (Supplementary Figure S3). Detailed information on the performance of the individual approaches is given in Table 2 and Figure 4. To test the library for specificity regarding possible mis-identifications of other bacterial organisms, a set of 25 strains from five commonly encountered bacterial species (*Escherichia coli*, *Pseudomonas aeruginosa*, *Staphylococcus aureus*, *Staphylococcus epidermidis*, and *Streptococcus pyogenes*) was generated. The isolates were first measured using a standard bacterial identification library (Compass Library MBT 8468 MSP, Bruker, Germany) and were confidently identified using automated spectra acquisition (Supplementary Table S1). Testing the same spectra against the final borrelia database yielded average score values of 0.643 and a maximum score value of 0.965, referring to no identification of borrelia in the sample.

**TABLE 2:** Performance of the generated databases (1,438 sum-spectral sets from 49 isolates of 13 species).

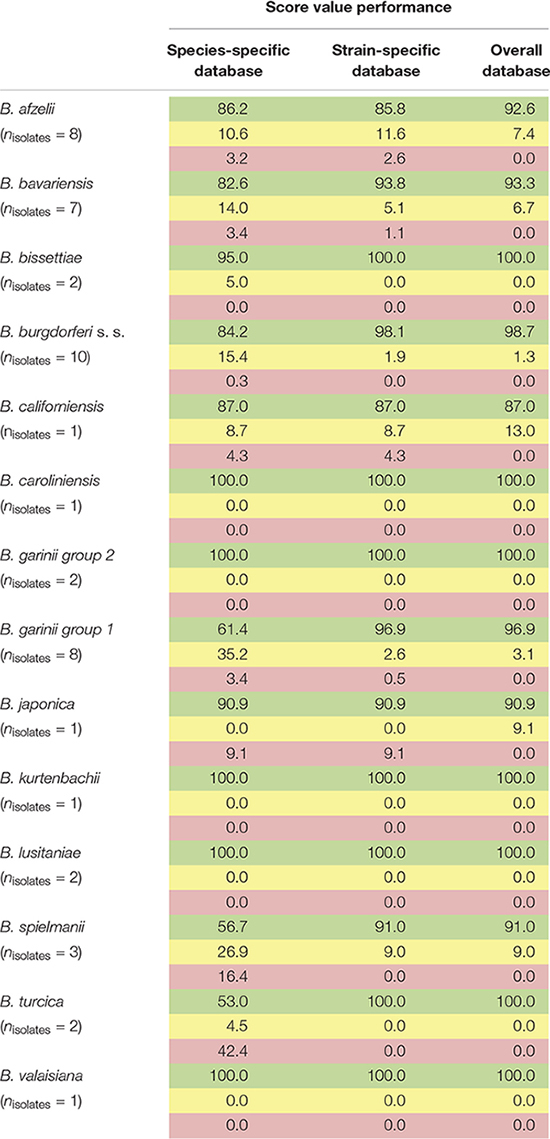

*The first column represents the species average database matches, the second column the strain average and the third column the combined database. Each value represents the percentage of the values that were obtained. Color code represents score values; green: >1.999, yellow 1.7–1.999; red < 1.7; score values in arbitrary units.*

## Discussion

In this study, we used 49 isolates of 13 *Borrelia* species to generate a MALDI-TOF MS database. Our sample set included one reptile-associated species and all assured human pathogenic *B. burgdorferi* species occurring in Europe and North America except *B. mayonii*, a human pathogenic species of limited distribution.

Two different extraction methods were tested for their suitability for *Borrelia* spp. differentiation. Our initial data showed that an extraction method frequently used for other microorganisms was not suitable for *Borrelia* spp. as bacterium-specific spectra were overlaid with peaks that likely represented artifacts generated by the protein-rich culture medium required for *Borrelia* spp. cultivation (Preac-Mursic et al., 1986; Pollack et al., 1993). We suspected that culture media specific mass peaks may lead to false positive results or misclassifications as well as low sensitivity for bacterial biomass. The media specific peaks may be due to insufficient washing. Using only medium without any further extraction did not yield detectable mass spectra, caused by its high salt content (data not shown). Thus, we tested the impact of medium on mass spectra, using the same extraction protocol on media alone that was also used for the targeted sample material. Indeed, similar spectra were observed with medium only samples as obtained for *Borrelia* containing samples (Supplementary Figure S1) further substantiating our suspicion that insufficient removal of medium by washing may lead to incorrect results.

The new sample preparation method developed within this study permits the generation of highly reproducible and pure MALDI-TOF MS spectra, even when using small numbers of spirochetes (<100,000). This is an important improvement, since *Borrelia* organisms are grown in liquid medium and it is not possible to collect uncontaminated bacteria from colonies as is the case for other bacteria that are grown on solid agar plates. Especially, the filter immobilization step of the organisms via vacuum suction is an important improvement as it immobilizes the borrelia organisms spread out on the filter membrane without agglomeration. This renders the cells accessible to washing as well as chemical extraction, increasing the yield of pure protein extracts. Further, this loss-less concentration technique allows subsequent filtering of larger volumes of spirochetes in liquid media with low densities onto one filter membrane increasing sensitivity. Another advantage of our method is that the extraction protocol can be performed on a 96-well platform allowing processing of many samples in parallel. Application on the target can be performed with the standard layering technique and matrix (HCCA) used in routine practice for bacterial identification, thus not requiring any changes in the workflow of the laboratory.

The ultimate step for sample preparation beyond what is presented here would be the sensitive direct detection of *Borrelia* from tissue biopsy materials of patients. This is currently only successful with PCR based techniques and even there, sensitivity is low. *Borrelia* culture from tissue is possible although often not routinely performed. Cultures growing borreliae can be identified with the protocol described here. Extraction directly from patient tissue seems complicated as bacterial loads are low and extensive contaminations due to patient tissue residues are expected in the sample even after digest/homogenization. Here, relapsing fever borreliae reaching high densities in peripheral blood might be an exception with sufficient amounts of bacteria in an easy to lyse matrix (blood) greatly facilitating the direct detection. We hope our protocol will advance the borrelia sample preparation on step further in the direction of working directly on patient material.

Our data demonstrates that differences between several isolates of one *Borrelia* species were considerable. This could be reliably reproduced. On the one hand, this offers the potential for strain/isolate typing and surveillance of long-term cultures in the laboratory, but constitutes on the other hand a problem for the generation of a database for identification of unknown isolates. Thus, we generated the database using multiple spectra of the individual isolates, as well as the sum spectra of all the strains within one species. Using only averaged species-spectra resulted in reliable identification at an average of 85.5% over all 13 species (*B. garinii* divided into two groups resulting in 14 groups). This is not a sufficient performance for routine application. Thereby, species with only one isolate showed better identification rates than species where multiple isolates were included. This is due to spectrum variability between the strains. To improve identification, we used the averaged spectra of all the individual strains for the database. Overall performance increased dramatically to 96.0% overall score equal or larger than score 2.0. However, still some measurements were below the score value of 1.7 and thus produced inconclusive results. By combining both approaches, we were able to obtain overall average identification rates with score ≥ 2.0 in 96.5%, while no measurements below score 1.7 were observed (Table 2 and Supplementary Figures S3, S4). Thus, identification rates can be considerably improved by including a wider range of strains, as well as the average spectrum of each species. This effect was most pronounced with species that consist of a wide variety of different isolates, which in turn were included in the analysis (Supplementary Figure S3). This also means that publications relying on a small number of isolates representing each strain might produce false trust in the overall real-life performance of the respective library and therefore these analyses should be interpreted with caution. Cluster analysis based on all the isolate-specific spectra demonstrates spectral homology of most strains of one species, proving the feasibility of this identification approach. The cluster analysis is surprisingly congruent with the phylogenetic relationship of the organisms based on MLSA data (representing the core genome) as well as the identification database performance (Table 2 and Figures 3, 4). This highlights the prospect of MALDI-TOF MS to serve as a tool for isolate/strain typing at an ultra-rapid speed combined with low costs. Surprisingly, in the cluster analysis two isolates, PLa and PFe (*B. garinii* group 2), clustered repeatedly separately from the bulk of *B. garinii* isolates, although PLa represents ST245 just as PKi, and PFe was typed to be ST244 as is PBr (see Figure 3). The exact reason for this is currently unknown, but is likely related to differences in protein expression patterns (as those are assessed by MALDI-TOF MS). One could speculate that this reflects differences in plasmid presence in these *B. garinii* strains. Further research into the plasmid content of these strains will be required to shed light on this. However, there is yet no established system to evaluate mass based strain typing. As the mass profile measures the abundance and mass of peptides and proteins detected in the respective sample, it may be prone to mutations changing the protein mass, as well as regulation phenotypes changing the protein patterns expressed in the individual strains. It is known that *Borrelia* spp. change the expression patterns of proteins during host or vector phases of their life cycle (Radolf et al., 2012; Caimano et al., 2019) and many of the involved genes are located on plasmids (Casjens et al., 2012, 2017). Research into expression patterns of proteins of *Borrelia* spp. has shown that genes may be switched on and off *in vitro* (Ojaimi et al., 2003; Iyer et al., 2015; Wu et al., 2015). To this point, the gene regulation and protein expression pattern *in vitro* differs from that in the host or vector and may change due to temperature or other external cues (Stevenson et al., 1995; Fingerle et al., 2000; Iyer et al., 2015). Thus, the spectra can in part reflect also regulation phenotypes. For most bacteria, this is not dramatic, as the protein spectra measured by MALDI-TOF MS largely represent smaller cationic ribosomal proteins, which are not heavily/significantly regulated and are highly conserved. Given the variability of the spectra observed in *Borrelia* spp., such proteins seem to contribute less to the overall MALDI-TOF MS spectrum in the genus *Borrelia* than they do in other species such as *E. coli* (Sohail, 2004).

It was encouraging to observe, that the accuracy of species and isolate identification was so high, for a majority of species and strains it is >95%, although the individual isolates had been acquired at different times from different sources and had been kept in *in vitro* culture for different duration and with different numbers of passages. Specificity of the library was assessed using 25 isolates from five different other bacterial species (Gram negative Rods and Gram positive cocci). The spectra of these isolates resulted in confident identification using a commercially available library, the same spectra did, however, not show any homology to *Borrelia* spectra, resulting in average score values of 0.643 (maximum 0.965, minimum 0.329) when analyzed using the final *Borrelia* library version. This demonstrates the high specificity of the library for *Borrelia* (Supplementary Table S1).

Taken together, we describe a novel, fast and sensitive sample preparation protocol for *Borrelia* spp. identification using MALDI-TOF MS, providing reproducible and pure spectra with only minimal influence of medium peaks from very small numbers of bacteria. The strain collection used represents the most important human pathogenic Lyme borreliosis species that occur in Europe and North America, i.e., *B. afzelii*, *B. bavariensis*, *B. burgdorferi* s.s., *B. garinii*, and *B. spielmanii*, the non-human pathogenic species *B. valaisiana* as well as species with unknown pathogenicity. It is the most comprehensive database of MALDI-TOF MS spectra of *Borrelia* spp. published so far. We also demonstrate the use of the mass data for phylogenetic studies using larger sets of borrelia strains. This technique might also advance capabilities toward the eventual analysis directly from patient material using mass spectrometric techniques.

## Data Availability Statement

The raw data supporting the conclusion of this article will be made available by the authors, without undue reservation, to any qualified researcher.

## Author Contributions

AW and A-CN-C designed the study, performed the extractions, mass spectrometric measurements and data analysis, and wrote the manuscript. VF and GM provided the strains, performed the genetic analysis, and wrote the manuscript. RS, EO, and SU performed the mass spectrometric measurements and extractions, and critically reviewed the manuscript.

## Conflict of Interest

The authors declare that the research was conducted in the absence of any commercial or financial relationships that could be construed as a potential conflict of interest.

## Footnotes

1 https://pubmlst.org/borrelia
