# Supplementary material for: A Novel Rapid Sample Preparation Method for MALDI-TOF MS Permits Borrelia burgdorferi Sensu Lato Species and Isolate Differentiation
Source: Front Microbiol. 2020 Apr 21;11:690. doi: 10.3389/fmicb.2020.00690 (PMC7186393; doi:10.3389/fmicb.2020.00690)
Supplement: Supplementary file 1 [file Data_sheet_1.pdf]

# *Supplementary Material*

## 1 Extraction protocol

The primary extraction protocol for samples from BSK-H medium was performed as recommended by Bruker according to the "ethanol formic acid extraction" protocol (Bruker Daltonics) and previous in-house studies (Rettinger et al., 2012). Each assay was repeated three times (technical replicates n=3) on different days (biological replicates) to proof the reproducibility. All centrifugation steps were done with 20800 *g (14,000 rpm) for 10 minutes at room temperature (RT). First, each culture was centrifuged; subsequently, the supernatant was removed, followed by a washing step with 1 mL distilled water. After resuspending the pellet, the culture was centrifuged again. The supernatant was removed, the pellet was resuspended in 300 µL distilled water and 900 µL of 96% ethanol (Roth, 96%, Ph.Eur., reinst., Karlsruhe, Germany) were added, respectively. Each tube was centrifuged for the third time. The supernatant was removed, followed by 10 minutes air-drying of the pellet. After evaporation of the ethanol/water mixture (v/v), 10 µL of formic acid (Merck, 98–100%, Darmstadt, Germany) and after resuspension, 10 µL of acetonitrile (Fluka Sigma-Aldrich, Munich, Germany) were added. Each tube was finally centrifuged for 2 minutes. One microliter of the clear supernatant was pipetted on a spot of a cleaned polished steel MSP-96 target (Bruker Daltonics) followed by air-drying. For calibration, the Bruker Bacterial Test Standard (BTS) (Bruker Daltonics) was spotted twice on each target at two defined positions. Finally 1µL of a saturated α-cyano-4-hydroxy-cinnamic acid matrix solution [HCCA portioned 2.5 mg (Bruker Daltonics) adding 250 µL "organic solvent" (50% acetonitrile, 47.5% ultra-pure water and 2.5% triflouracetic acid; Fluka Sigma-Aldrich) was transferred on each spot. The target was air-dried and directly introduced to the MALDI-TOF mass spectrometer (Microflex-LT, Bruker Daltonics).

The sample preparation was performed simultaneously with pure culture media (BSK-H) to assess the influence of media to the spectra generated for the *Borrelia* spp. For spectrometer settings and calibration see Rettinger et al. (2012). Spectra were created in the automatic mode with initial and maximal laser power set between 30-40%. Each spot on the target was measured three times with the Flex control software (version 3.4, Bruker Daltonics) using the spiral-small raster.

ClinProTools software (version 3.0, Bruker Dalontiks) was used for reviewing the BSK-H spectra. Spectra of pure BSK-H and spectra of the *Borrelia* spp. cultured in BSK-H were compared with each other to test similarities.

# 2 Supplementary Figures

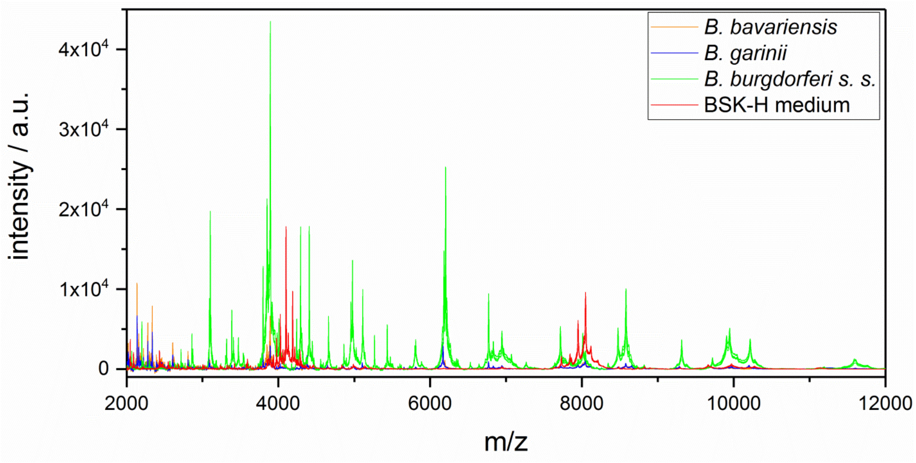


**Supplementary Figure S1.** Sum spectra of three different *Borrelia* spp. (yellow: *B. bavariensis*, blue: *B. garinii*, green: *B. burgdorferi* s.s.) and BSK-H medium (red) following the non-optimized washing protocol [a.u.: arbitrary units].

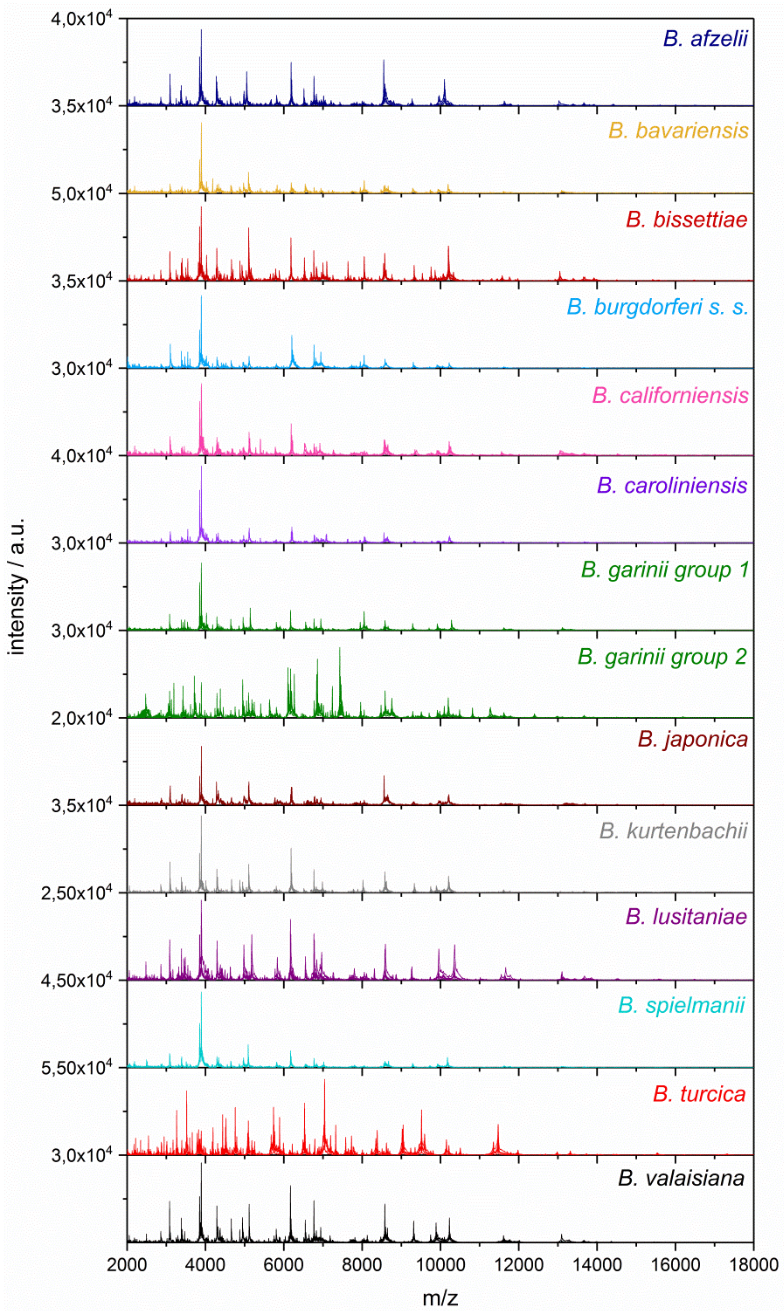


**Supplementary Figure S2.** Representative MALDI-TOF MS spectra of 13 *Borrelia* species (*B. garinii* is split into mass group 1 and 2) used in this study, performed with the new membrane based washing protocol (quintuplicates plotted in each spectrum) Bruker Daltroniks [a.u.: arbitrary units].

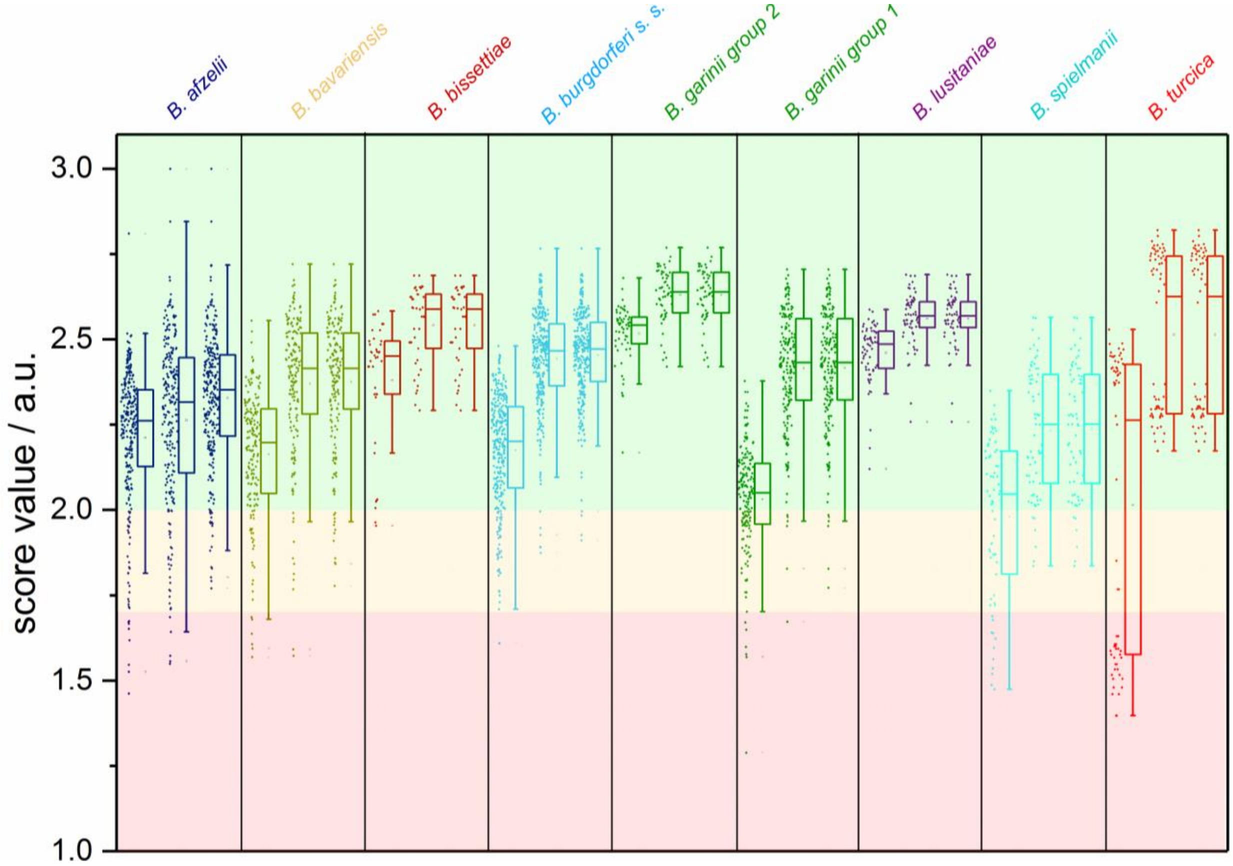


**Supplementary Figure S3.** Score results [a.u.: arbitrary units] of the species with multiple isolates within the study. *B. garinii* is divided and treated as two separate mass groups (1 and 2) for the analysis. The leftmost dataset in each column represents the performance of a database using average spectra of all isolates. The second dataset from the left uses only the individual strains as database entry. The third dataset per column represents the database with the best performance, using both, all individual strains as database entries as well as the species average spectra. Especially, the identification of *B. turcica*, *B. spielmanii*, *B. garinii* mass group 2 and *B. burgdorferi* s.s. benefits most from the strain database. *B. afzelii* and *B. burgdorferi* s.s. improve most by adding the average spectra in the combined database. These species are those with most different tested isolates.

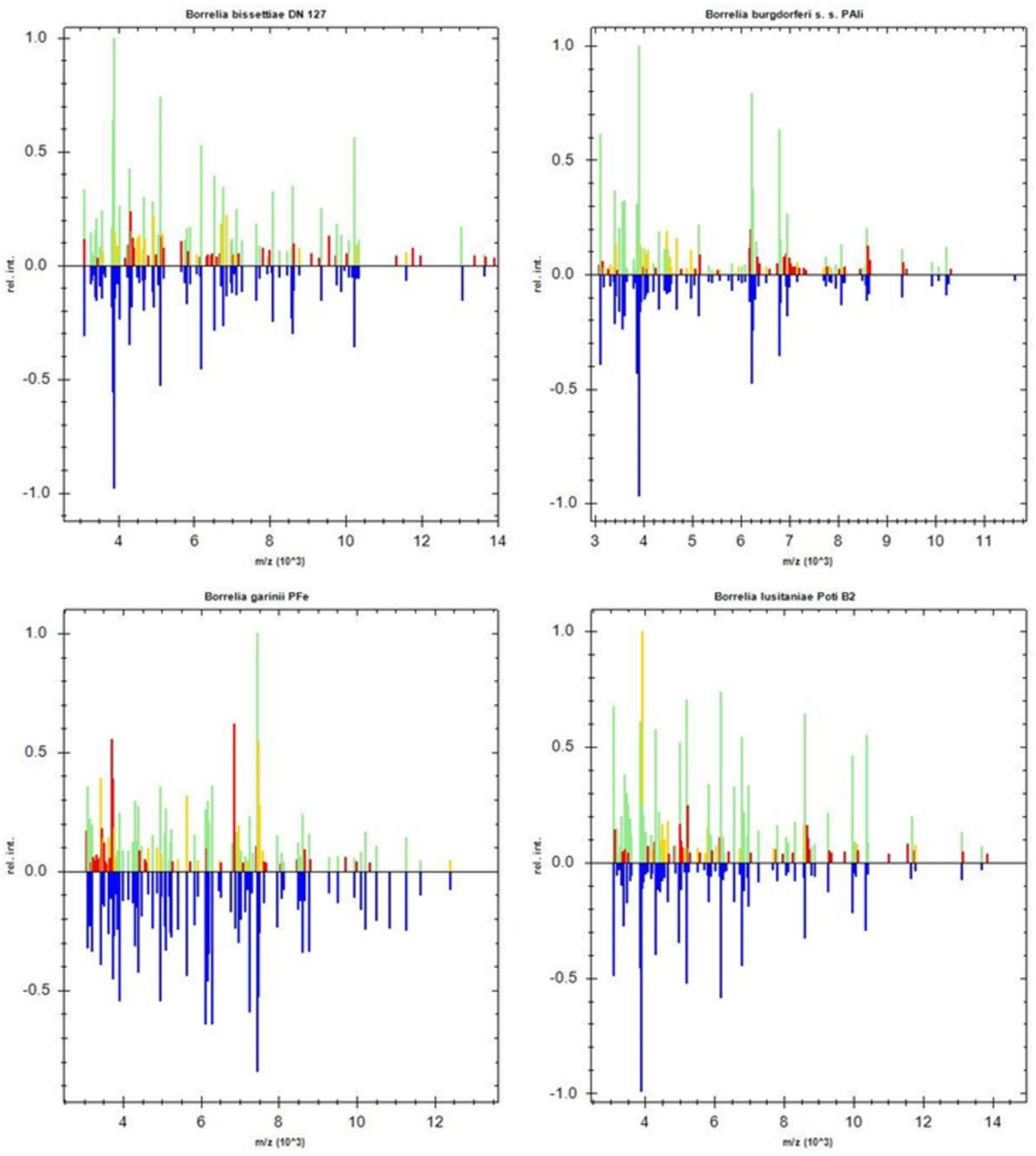


**Supplementary Figure S4.** Exemplary identification of *B. bissettiae* DN 127, *B. burgdorferi* s.s. PAli, *B. garinii* PFe and *B. lusitaniae* Poti B2. The upper part of each graph represents the normalized peak list spectrum of the unknown sample and the lower part each represents the peak list spectrum of the corresponding MSP as saved in the database.

| Organism | Score Biotyper-database | Organism | Score Borrelia-database | Organism |
|---|---|---|---|---|
| *E. coli* | 2.330 | *E. coli* | 0.801 | *B. bavariensis* |
| | 2.266 | *E. coli* | 0.599 | *B. californiensis* |
| | 2.362 | *E. coli* | 0.423 | *B. burgdorferi* s.s. |
| | 2.305 | *E. coli* | 0.623 | *B. bavariensis* |
| | 2.374 | *E. coli* | 0.705 | *B. spielmannii* |
| *P. aeruginosa* | 2.246 | *P. aeruginosa* | 0.761 | *B. californiensis* |
| | 2.264 | *P. aeruginosa* | 0.722 | *B. spielmannii* |
| | 2.193 | *P. aeruginosa* | 0.692 | PLa |
| | 2.325 | *P. aeruginosa* | 0.853 | *B. carolinensis* |
| | 2.161 | *P. aeruginosa* | 0.449 | *B. carolinensis* |
| *S. aureus* | 2.320 | *S. aureus* | 0.551 | *B. burgdorferi* s.s. |
| | 2.324 | *S. aureus* | 0.533 | *B. bavariensis* |
| | 2.126 | *S. aureus* | 0.635 | *B. garinii* |
| | 2.212 | *S. aureus* | 0.400 | PBabu |
| | 2.347 | *S. aureus* | 0.329 | *B. bavariensis* |
| *S. epidermidis* | 2.176 | *S. epidermidis* | 0.788 | *B. bavariensis* |
| | 2.127 | *S. epidermidis* | 0.720 | *B. valaisiana* |
| | 2.164 | *S. epidermidis* | 0.727 | *B. bissettiae* |
| | 2.083 | *S. epidermidis* | 0.556 | *B. burgdorferi* s.s. |
| | 1.951 | *S. epidermidis* | 0.685 | *B. valaisiana* |
| *Strep. pyogenes* | 2.227 | *Strp. pyogenes* | 0.634 | *B. turcica* |
| | 2.258 | *Strp. pyogenes* | 0.762 | *B. turcica* |
| | 2.145 | *Strp. pyogenes* | 0.828 | *B. burgdorferi* s.s. |
| | 2.159 | *Strp. pyogenes* | 0.332 | *B. turcica* |
| | 2.168 | *Strp. pyogenes* | 0.965 | *B. garinii* |
| Average | 2.225 Score | | 0.643 Score | |
| Confident ID | 96% | 24/25 | 0% | 0/25 |

**Supplementary Table S1.** Five isolates of *Escherichia coli* (*E. coli*), *Pseudomonas aeruginosa* (*P. aeruginosa*), *Staphylococcus aureus* (*S. aureus*), *Staphylococcus epidermidis* (*S. epidermidis*) and *Streptococcus pyogenes* (*Strep. pyogenes*) were cultured and analyzed. Thereby, the spectra were identified using the Biotyper Software 3.0 with Compass Library MBT 8468 MSP. Isolates were identified correctly in 96% of cases with an average score of 2.225. The same spectra run against the

*Borrelia* database yielded no misidentification and an average score value of 0.643, demonstrating the specificity of the library against other bacterial strains commonly encountered.

## References

Rettinger, A., Krupka, I., Grunwald, K., Dyachenko, V., Fingerle, V., Konrad, R., et al. (2012). Leptospira spp. strain identification by MALDI TOF MS is an equivalent tool to 16S rRNA gene sequencing and multi locus sequence typing (MLST). *BMC Microbiol* 12**,** 185. doi: 10.1186/1471-2180-12-185.
